# Applying virtual reality to sail education: an innovative strategy to enhance knowledge learning for student novices

**DOI:** 10.3389/fpsyg.2025.1498725

**Published:** 2025-02-26

**Authors:** Shan Zhao, Fa Ji

**Affiliations:** ^1^School of Physical Education, Qingdao University, Qingdao, China; ^2^Development Center for Water Sports, Qingdao University, Qingdao, China

**Keywords:** sailing, virtual reality, novice, physical education, randomized controlled trial

## Abstract

**Background:**

Sailing has been proved beneficial for physical and mental health promotion, which has made it a prevalent sport among children and adolescents. Nevertheless, the existing pedagogical strategies cannot satisfy students’ demands for learning. To bridge this gap, virtual reality (VR) is considered an innovative approach to addressing the challenges in sailing education.

**Objective:**

The study aimed at designing an education program to examine effectiveness of VR technology in sail knowledge learning.

**Methods:**

University students with no prior experience or knowledge in sailing were recruited and randomly allocated to either experimental group (*N* = 32) using VR in lectures and self-practice or control group (*N* = 34) attending traditional lectures. The sail education program consisted of 12 sessions over 6 weeks. Sailing knowledge test was developed from an official manual by American Sailing Association to assess outcomes of learning in overall performance, sailboat structure, sailing skills, and navigation rules. The Situational Motivation Scale (SIMS) was used to assess the constructs of intrinsic motivation, identified regulation, external regulation, and amotivation. A 2 × 2 MANOVA was conducted for statistical analysis.

**Results:**

Both groups improved accuracy rate after the program, with significant time effects in all outcome measures. Particular advantages of VR were identified in facilitating knowledge learning on sailing skills and navigation rules. Significant interaction effects suggest that application of VR induced greater improvement than traditional lectures. Comparable performance between the two groups was found in sailboat structure, with non-significant results in group effect and interaction effect. What’s more, VR could better improve students’ intrinsic motivation and identified regulation while better reduce external motivation and amotivation in sailing lessons.

**Conclusion:**

The findings verified feasibility of applying VR to promote sail education for student novices. To enhance effects of VR in teaching and learning, course design should focus on reflective connections between knowledge and experience, which stimulate students in active, engaging, and insightful learning.

## 1 Introduction

Long ago, sails carried humans across the oceans in quest of discovery, wealth, and power. As time goes by, it has been discovered that sailing carries great value in personal development and education. A multinational qualitative study showed that sail training is a powerful educative experience having a special benefit in developing social confidence for young people (McCulloch et al., 2010). To further explore the impact of sailing on children, researchers conducted a qualitative study of a small sailing program with 38 children (9–13 years old). Semi-structured interviews and focus groups on children, teachers, and sailing instructors identified the main benefits of dinghy sailing in physical and mental health, development of key life skills, self-esteem, and academic performance (Cotterill and Brown, 2018). As well, a quasi-experimental study involving 147 adolescents found that even a short sail training activity can significantly improve participants’ Social and Competence self-concept (Capurso and Borsci, 2013). A comprehensive meta-analysis indicated that sailing, as one of the adventure sports, has a positive impact on leadership, self-concept/self-esteem, locus of control, interpersonal attitudes, physical fitness, and environmental awareness (Hattie et al., 1997). While the sport has many benefits, factors such as high cost, complicated skills, and geographical restraints make it a niche sport whose participants are mainly professional athletes. To provide more people with the opportunity to access and learn the sport, experts have suggested promoting sailing into schools as a formal education, thereby breaking down geographical and economic constraints (Schmitt et al., 2020). This approach can ensure planned, systematic, comprehensive, and large-scale lessons, effectively selecting and developing sailing talents. According to the 5-year plan (2021–2025) released by the Chinese government, it is expected that by 2025, in Qingdao, the number of sailing-featured schools will be increased to 132 and the number of students learning basic sailing knowledge will reach 22,000 (Qingdao, 2021).

However, designing and implementing sailing lessons under physical education (PE) settings remains challenging for practical reasons. Unlike athletes with rich knowledge and skillful performance over longitudinal training, students in PE classes have no or limited previous sailing experience. Improving the skill proficiency of the novices by sailing on the sea may raise concerns about time efficiency, cost, and safety. For the programs that begin with theory learning, the abstract knowledge can be difficult for novices to understand without hands-on practice. To better satisfy students’ demands for more active, engaging, and insightful learning, there are researchers innovating pedagogical strategies. An experimental study involving 80 students found that the flipped classroom teaching model can greatly enhance participants’ satisfaction level and academic knowledge of sailing (Caraballo Vidal et al., 2024) while it created a huge workload for teachers and students to find textual information, thus reducing the effectiveness of instruction. Another quantitative study showed that teaching races for understanding (TRFU) can be used in sailing to improve students’ capacity to reflect and connect theoretical knowledge with their motor performance in the race (Morales-Belando and Arias-Estero, 2017). Yet, this program was limited by space and weather and not suitable for large-scale teaching. Therefore, helping more students visualize task-related situations and corresponding operations in safe, interactive, and controlled environments is an effective way to solve the existing problems.

Virtual reality (VR) is considered an innovative approach to addressing the challenges in sailing education (Ji et al., 2023). This technology provides a powerful tool for implementing situated learning perspective and cognitive theory of multimedia learning. It can present theoretical knowledge in the form of 3D practical scenarios (Plotzky et al., 2021) or simulated lifelike learning situations allowing users to immersively interact with virtual objects, thus reducing cognitive load and stimulating imagination to assist the mind’s capacity to conceptualize. In addition, VR is the perfect complement to other practical classes and enables faster skill acquisition and better retention. On the one hand, characteristics such as immersive experience, first-person perspective, and self-paced task constraints make VR technology feasible for accelerating the transition from procedural knowledge to practical skills, which has led to its wide application in many sports. For example, in training baseball batting, VR can simulate real batting situations and provide adaptive baseball training involved performance-based adjustments of pitch speed, pitch type, and location, to improve athletes’ perceptual-motor skills (Gray, 2017). Similarly, in basketball offensive tactics (Tsai et al., 2022) and table tennis (Oagaz et al., 2022) technical training, VR can also provide realistic training environments to help athletes improve their tactical understanding and reaction speed, thus showing greater adaptability and competitiveness in the face of complex real-world tasks. On the other hand, in a safe virtual environment, students can repeat the actions that are not easy to perform in practical situation as many times as they like, thus building muscle memory and leading to longer retention. Furthermore, VR technology has been shown to be effective in increasing student motivation (Garris et al., 2002), interaction, and active engagement (Roussou, 2004) in other educational settings and subjects.

The unique features of sailing compared to other sports, such as intricate sailboat structures, complex navigation rules, and dynamic environmental factors, make it an ideal candidate for VR-based instruction. The immersive and interactive characters of VR can potentially enhance students’ understanding and retention of sailing concepts, skills, and strategies. Although some researchers have recognized the potential of VR-based sailing education in teaching practice (Wu et al., 2020), the scientific community still lacks an in-depth understanding of how VR technology can specifically promote the effectiveness of sailing knowledge learning in a physical education (PE) setting. Currently, relevant studies still remain in the theoretical exploration stage, lacking rigorous experimental design and systematic effect evaluation to accurately judge the practical application value of VR technology in sailing education. To fill this research gap, the current study develops a VR-based sailing education program and designs a randomized controlled trial to evaluate its effectiveness compared to traditional sailing lectures. Academic performance (which encompasses overall sailing knowledge, sailboat structure understanding, sailing skills, and navigation rule knowledge) and motivation (including intrinsic motivation, identified regulation, external motivation, and amotivation) were used as indicators of learning outcomes in this study, because the former reflects students’ mastery of sailing knowledge and skills, while the latter underlies behavior and can influence persistence, effort, and overall success in learning (Ryan and Deci, 2020). Our primary focus was on testing the following core hypotheses:

*H1*: Compared with the traditional approach, VR technology can be more effective in improving student novices’ overall sailing knowledge, sailboat structure, sailing skill, and navigation rule knowledge.

*H2*: Compared with the traditional approach, VR technology can be more effective in improving student novices’ autonomous motivation.

By testing these hypotheses, the study aims to contribute valuable insights into the effectiveness of VR-based sailing education and its potential to improve learning outcomes in PE settings. The results of the study can inform educators and researchers about the potential benefits of integrating VR technology into sailing education, and contribute to the development of more effective and engaging teaching methods in the field, thereby promoting the popularity of sailing and enabling more individuals to reap its benefits.

## 2 Materials and methods

### 2.1 Participants

A total of 68 undergraduate students from Qingdao University in China were recruited as participants (age = 18.42 ± 1.084; female = 36, male = 32). The sample size was determined by a prior power analysis at *α* level of 0.05, power (1—*β*) of 0.80 (Faul et al., 2009), and 10% of drop rate (Zhang et al., 2023). Eligible participants should meet the following criteria: (1) good health and free of vertigo, (2) normal or corrected to normal vision, (3) no sailing knowledge and experience, and (4) no schedule conflicts with the class. Recruitment information was predominantly disseminated by flyers and social media. Additional approach included emails and presentations at classes. This study was approved Ethics Committee of Qingdao University, and the collected data were processed anonymously. Written informed consent was obtained from all participants.

The participants were randomly allocated to experimental group (EG, *N* = 34) and control group (CG, *N* = 34) by a computer-based random number generator. Before the intervention, however, two participants in EG missed the pre-test and the first lesson because of flu, resulting in 32 participants in EG. Independent sample *t*-test was conducted to identify potential between-group differences at the baseline. The results indicated that all *p*-values were greater than 0.05, suggesting no significant difference in prior knowledge between both groups (Table 1).

**Table 1:** Differences between groups at the baseline.

Category	EG (M ± SD)	CG (M ± SD)	*p*-value
Sailing knowledge (%)	45.72 ± 7.19	42.03 ± 9.02	0.07
Sailboat structure (%)	31.25 ± 10.70	28.24 ± 11.41	0.27
Sailing skills (%)	46.06 ± 9.57	44.85 ± 11.42	0.64
Navigation rules (%)	62.69 ± 14.89	62.50 ± 18.46	0.96
Intrinsic motivation	16.44 ± 5.91	14.76 ± 5.67	0.25
Identified regulation	16.34 ± 4.96	14.76 ± 6.14	0.26
External regulation	17.72 ± 6.42	20.18 ± 3.95	0.18
Amotivation	16.56 ± 7.47	19.38 ± 4.29	0.07

EG, experimental group; CG, control group; M, mean; SD, standard deviation.

### 2.2 Study materials and apparatus

The sailing courses were designed in accordance with the book, Sailing Made Easy, which is American Sailing Association’s official manual of basic sailing standards (American Sailing Association, 2010). Content of chapters 1, 3, 5, 6, and 7 was taught in six sessions for both groups. Specifically, critical points covered in the sessions included the points of sail, structure of a sailboat, momentum control, turning the boat, docking, and safe sailing, etc. A prominent advantage of the book was the review questions presented at the end of each chapter, which provided essential references to develop sail knowledge test.

The VR headset applied to the intervention was Meta Quest 2 head mounted display (HMD). This device has raised researchers’ interest because of its portability and convenience to use. It is a wireless standalone device which is independent of a computer and external tracking system (Trinidad-Fernández et al., 2023). The VR courses were conducted by means of a virtual sailing game, MarineVerse Cup (Virtual Reality Sailing Pty Ltd., Melbourne, Australia). Utilizing VR headsets, this fully immersive app can provide extensive 3D sailing tutorials on different sailboat models, navigation rules, and sailing skills which provide a good match with the textbook content. Table 2 summarizes the tutorial modules of the VR game in correspondence to the book chapters.

**Table 2:** Tutorial modules of the VR game in correspondence to the book chapters.

Tutorial modules	Book chapters
Sailboat structure	Chapter 1
Parts of sailboat	
Sailboat’s Rig	
On-board orientation	
Sailing skills	Chapters 3, 5, and 7
Tacking	
Jibing	
Using spring lines	
Docking	
Navigation rules	Chapter 6
Buoys and marks	
Stand-on and give-way rules	

### 2.3 Outcome measures

#### 2.3.1 Sailing knowledge test

To assess the prior knowledge before the intervention (i.e., pre-test), and achievement following the intervention (i.e., post-test), an online sailing knowledge test was developed with the following procedures. Firstly, three experts were invited to adapt 40 multiple-choice questions from textbook to construct the initial test. Secondly, 14 respondents who had attended the traditional lectures were invited to participate in the pilot test. Based on classic test theory (CTT), we analyzed that the test has satisfactory validity and reliability. The discrimination indices of all 40 items ranged from 0.33 to 0.86, which means there was no need for further revision because greater than 0.30 was considered acceptable (Lien, 1971). The difficulty indices with a mean value of 0.59 clustering around 0.50, support this test has good discrimination power (Brookhart and Nitko, 2011). The reliability coefficient of Cronbach’s alpha was 0.732 which was within the acceptable level of reliability (range 0.70–0.80) (Nunnally and Bernstein, 1994). Eventually, all 40 questions were included in the final paper. Accuracy rate was the measure of performance, and a higher rate implies a favorable learning outcome.

#### 2.3.2 Situational motivation scale

In order to understand students’ current (or state) self-regulatory processes in different sailing classes, the English version of Situational Motivation Scale (SIMS) (Guay et al., 2000), which has good reliability and factorial validity in a PE context among adolescents (Østerlie et al., 2019), was used in this current study. Based on the self-determination theory (SDT) (Deci and Ryan, 2000), the SIMS assesses a more diverse range of types of motivation, namely intrinsic motivation, identified regulation, external regulation, and amotivation, which significantly overcomes the limitations of traditional measures, such as the free-choice measure and self-report questionnaires (Guay et al., 2000). This scale comprises a total of 16 items, systematically organized into four subscales, each containing four items. Notably, the Cronbach’s alpha values for these four subscales ranged from 0.77 to 0.95, indicating robust internal consistency and reliability. Respondents were asked to indicate their level of agreement on this 7-point Likert scale, ranging from 1 (strongly disagree) to 7 (strongly agree). Their chosen number was then converted into a corresponding score, with all items scored positively.

### 2.4 Study design and procedures

**Figure 1:**
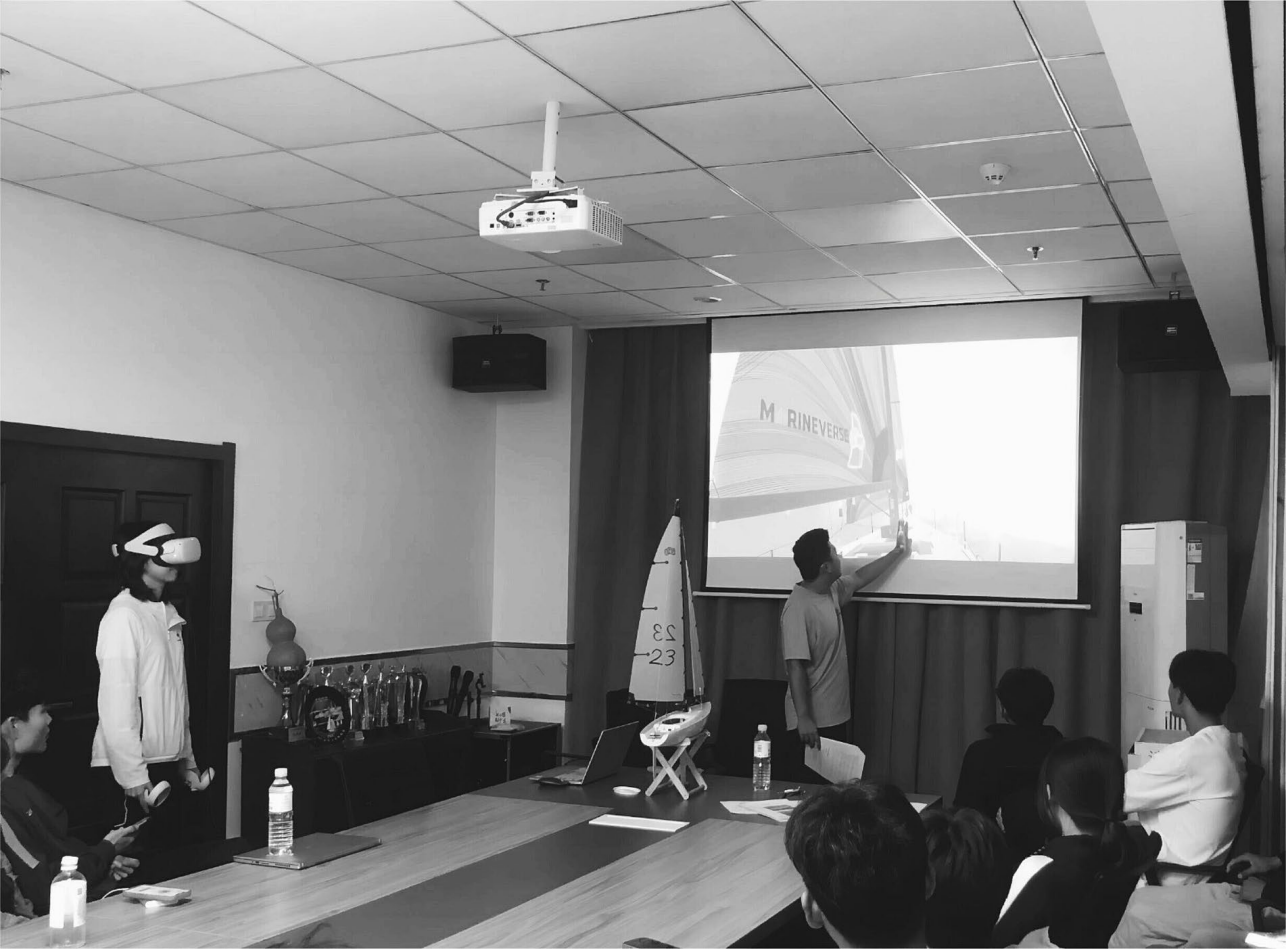
Demonstration of the VR-based lecture.

The current study adopted a randomized controlled design for 12 h of sail knowledge learning. EG completed 6 h of VR lectures and 6 h of self-directed practice. In the VR sessions, an instructor gave a lecture while a teaching assistant collaborated by wearing the VR headset to present the first-person view of sailing (Figure 1).

Self-directed practice was arranged as a review session after each lecture. Students made an appointment with a research assistant according to availability of the device. By taking advantage of after-school hours, the students practiced the content of previous lecture in a VR-simulated environment. Each review session was conducted under supervision of a teaching assistant who was responsible to provide instructions and technical support during practice.

Control group provided novices with only lecture-based classes for 12 sessions over 6 weeks. In the lectures for CG, the same content was covered as EG. The instructor used slides, pictures, and videos to assist in understanding.

**Figure 2:**
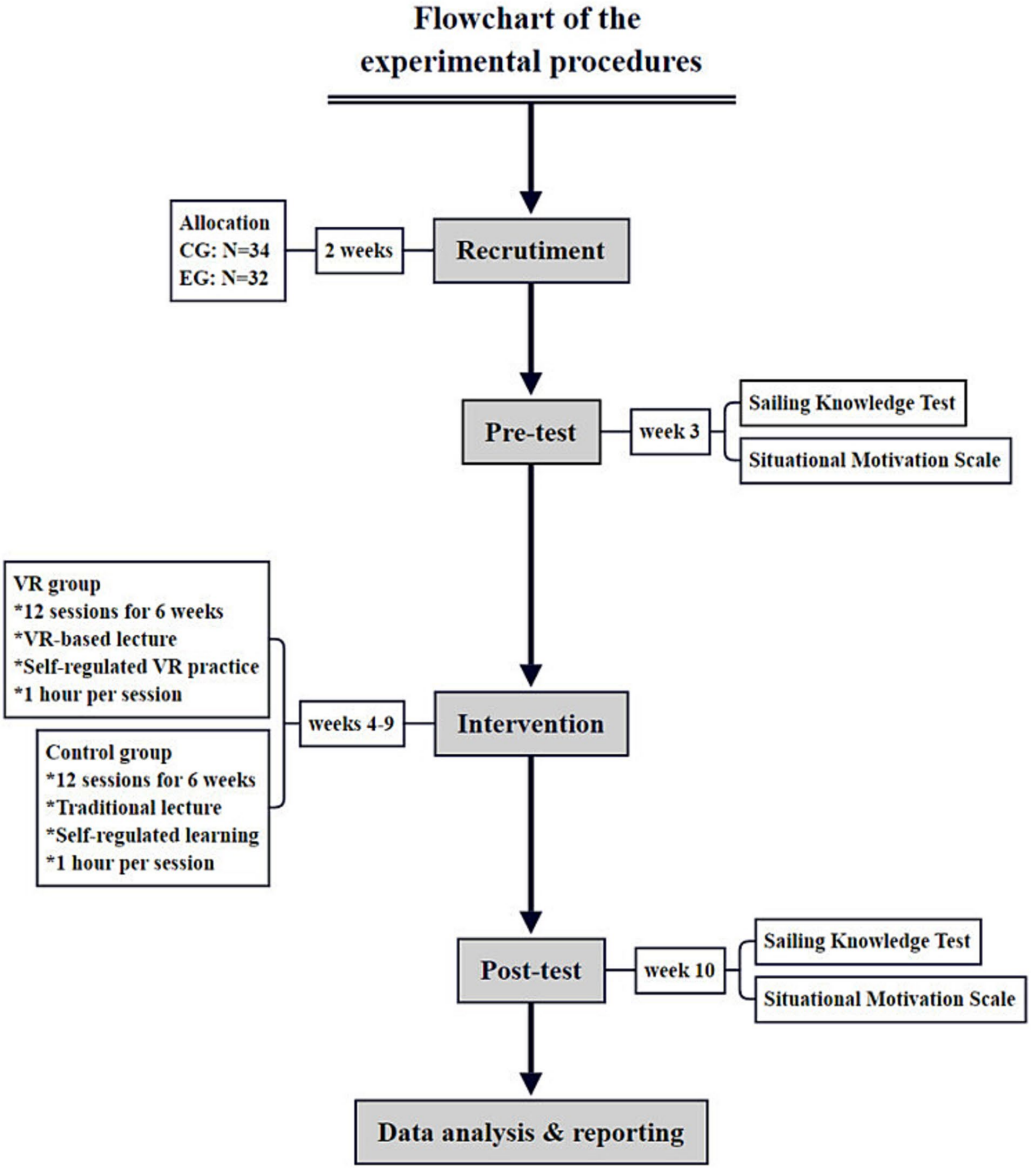
Flowchart of the experimental procedures.

All participants completed the sailing knowledge test and SIMS online within 45 min in pre- and post-test. To ensure integrity in the tests, behaviors such as page refreshes, copy and paste, and communication with others were not allowed during the test. After each test, the system automatically collected the questionnaires while ensuring data security and privacy protection. Figure 2 illustrates procedures of the research project.

### 2.5 Data analysis

A 2 × 2 repeated measures multivariate analysis of variance (MANOVA) was conducted to analyze the main effect of time, intervention, and the interaction effect on sailing knowledge and situational motivation. Independent variables include both between-group factor (Group: EG vs. CG) and within-group factor (Time: pre-test vs. post-test). Dependent variables in sailing knowledge are overall performance in sail knowledge test and individual categories of knowledge on sailboat structure, sailing skills, and navigation rules while in motivation are intrinsic motivation, identified regulation, external motivation, and amotivation.

Data points beyond three standard deviations from the mean were defined as outliers. Normality assumption was examined by the Shapiro–Wilk test. Levene’s test of equality of error variances was performed to verify homogeneity of variance assumption. Statistical significance was defined by the cutoff point of 0.05. Partial eta squared (*η*^2^) measured effect size, with 0.01, 0.06, and 0.14 defining small, moderate, and large effects (Cohen, 1988). All statistical analyses were conducted by SPSS 25.

## 3 Results

### 3.1 Effect of teaching methods on sailing knowledge

Significant improvement in overall sailing knowledge was found for both groups after the 6-week sail education program, which was evidenced by the main effect of time, *F*(1, 64) = 1162.52, *η*^2^ = 0.95, *p* < 0.001. It is worth noting the significant group effect, *F*(1, 64) = 27.43, *η*^2^ = 0.30, *p* < 0.001. Despite similar performance in the pre-test (EG: 45.72%, CG: 42.03%, *p* = 0.07), students taking VR-based lectures outperformed their counterparts receiving traditional lectures in the post-test (EG: 93.91%; CG: 84.19%, *p <* 0.001). The interaction effect also reached the significant level, *F*(1, 64) = 5.17, *η*^2^ = 0.08, *p* = 0.03, indicating that VR-based lectures induced greater improvement than traditional lectures.

The subsequent analyses focused on each category of the sailing knowledge. Specifically, knowledge regarding sailboat structure improved by the end of the program, which was substantiated by the significant main effect of time, *F*(1, 64) = 1027.70, *η*^2^ = 0.94, *p* < 0.001. Both EG (pre-test: 31.25%, post-test: 93.13%, *p* < 0.001) and CG (pre-test: 28.24%, post-test: 91.18%, *p* < 0.001) indicated significant improvement in the post-test. The main effect of group was non-significant, *F*(1, 64) = 1.69, *η*^2^ = 0.03, *p* = 0.20, indicating similar performance between the two groups. Additionally, interaction effect was non-significant, *F*(1, 64) = 0.08, *η*^2^ = 0.001, *p* = 0.79, suggesting comparable performance change over time.

Knowledge on sailing skills was learned after the program due to the significant main effect of time, *F*(1, 64) = 677.08, *η*^2^ = 0.91, *p* < 0.001. Both EG (pre-test: 46.06%, post-test: 94.03%, *p* < 0.001) and CG (pre-test: 44.85%, post-test: 81.55%, *p* < 0.001) showed a higher accuracy in the post-test. The main effect of group was statistically significant, *F*(1, 64) = 17.47, *η*^2^ = 0.21, *p* < 0.001. VR-based learning resulted in a favorable outcome to traditional lectures in the post-test (EG: 94.03%, CG: 81.55%, *p* < 0.001). The magnitude of improvement was greater in EG compared with CG, which was supported by a significant interaction effect, *F*(1, 64) = 12.00, *η*^2^ = 0.16, *p* = 0.001.

In terms of knowledge on navigation rules, significant results were identified in the main effect of time, *F*(1, 64) = 90.58, *η*^2^ = 0.59, *p* < 0.001, indicating a higher accuracy in the post-test. VR technology facilitated understanding of navigation rules. This can be substantiated by significant group effect, *F*(1, 64) = 7.98, *η*^2^ = 0.11, *p* = 0.01, and favorable performance associated with EG in the post-test (EG: 94.53%, CG: 82.72%, *p* < 0.001). A significant interaction effects was identified, *F*(1, 64) = 4.52, *η*^2^ = 0.07, *p* = 0.04, indicating that VR technology induced a greater improvement than traditional lectures (Table 3).

**Table 3:** Academic performance in sailing knowledge tests.

Category	Time	EG	CG	Two-way MANOVA		
		M ± SD	M ± SD	Time effect	Group effect	Interaction effect
Sailing knowledge (%)	Pre	45.72 ± 7.19	42.03 ± 9.02	*F* = 1162.52 *η^2^* = 0.95 *p <* 0.001	*F* = 27.43 *η^2^* = 0.30 *p* < 0.001	*F* = 5.17 *η^2^* = 0.08 *p* = 0.03
	Post	93.91 ± 5.08	84.19 ± 7.92			
Sailboat structure (%)	Pre	31.25 ± 10.70	28.24 ± 11.41	*F* = 1027.70 *η^2^* = 0.94 *p* < 0.001	*F* = 1.69 *η^2^* = 0.03 *p* = 0.20	*F* = 0.08 *η^2^* = 0.001 *p* = 0.79
	Post	93.13 ± 9.98	91.18 ± 12.00			
Sailing skills (%)	Pre	46.06 ± 9.57	44.85 ± 11.42	*F* = 677.08 *η^2^* = 0.91 *p* < 0.001	*F* = 17.47 *η^2^* = 0.21 *p* < 0.001	*F* = 12.00 *η^2^* = 0.16 *p* = 0.001
	Post	94.03 ± 6.37	81.55 ± 9.29			
Navigation rules (%)	Pre	62.69 ± 14.89	62.50 ± 18.46	*F* = 90.58 *η^2^* = 0.59 *p* < 0.001	*F* = 7.98 *η^2^* = 0.11 *p* = 0.01	*F* = 4.52 *η^2^* = 0.07 *p* = 0.04
	Post	94.53 ± 8.36	82.72 ± 12.32			

EG, experimental group; CG, control group; M, mean; SD, standard deviation.

### 3.2 Effect of teaching methods on motivation

Compared with traditional lectures, VR induced greater improvements in students’ intrinsic motivation and identified regulation but larger reductions in external motivation and amotivation.

In intrinsic motivation, the main effect of time was significant, *F*(1, 64) = 16.82, *η*^2^ = 0.21, *p* < 0.001. Both EG (pre-test: 16.44, post-test: 20.22, *p* = 0.003) and CG (pre-test: 14.76, post-test: 17.97, *p* = 0.009) showed a higher intrinsic motivation in the post-test. The main effect of group was also statistically significant, *F*(1, 64) = 4.97, *η^2^* = 0.07, *p* = 0.03. Further analysis found a higher level of intrinsic motivation associated with VR-based learning than traditional lectures in the post-test (EG: 20.22, CG: 17.97, *p* = 0.03). Additionally, interaction effect was non-significant, *F*(1, 64) = 0.11, *η*^2^ = 0.002, *p =* 0.74, suggesting comparable performance change over time.

The identified regulation indicated similar outcomes to the intrinsic motivation. The main effect of time was significant, *F*(1, 64) = 14.96, *η*^2^ = 0.19, *p* < 0.001, suggesting an overall improvement in the post-test. The group effect was also statistically significant, *F*(1, 64) = 4.48, *η^2^* = 0.07, *p* = 0.04. There was no significant difference between EG and CG in the pre-test (*p* = 0.26), but EG outperformed CG in the post-test at a statistically significant level (EG: 19.91, CG: 17.65, *p* = 0.04). The interaction effect was non-significant, *F*(1, 64) = 0.17, *η*^2^ = 0.003, *p =* 0.69, suggesting comparable performance change over time.

In external motivation, significant results were identified in the main effect of time, *F*(1, 64) = 13.23, *η*^2^ = 0.17, *p* = 0.001, as well as the main effect of group, *F*(1, 64) = 9.20, *η^2^* = 0.13, *p* = 0.003. No significant result was observed in interaction effect, *F*(1, 64) = 0.002, *η*^2^ < 0.001, *p* = 0.96. However, it is worth noting that the external motivation reduced in the post-test whether in the VR group (pre-test: 17.72, post-test: 14.19, *p* = 0.01) or the control group (pre-test: 20.18, post-test: 16.74, *p* = 0.01). Students in EG had lower external motivation than in CG after the sail education program (EG: 14.19, CG: 16.74, *p* = 0.04).

Consistent findings with the external motivation were identified in amotivation. The main effect of time, *F*(1, 64) = 13.91, *η*^2^ = 0.18, *p* < 0.001, and the main effect of group, *F*(1, 64) = 14.96, *η^2^* = 0.19, *p* < 0.001, were statistically significant. In contrast, the interaction effect was non-significant, *F*(1, 64) = 0.54, *η*^2^ = 0.008, *p* = 0.47. Further analyses found significant reductions in autonomous motivation after the intervention in both EG (pre-test: 16.56, post-test: 12.09, *p* = 0.003) and CG (pre-test: 19.38, post-test: 16.38, *p* = 0.04). No significant difference was found between EG and CG in the pre-test (*p* = 0.06). In the post-test, however, students in EG had lower autonomous motivation than their counterparts in CG (EG: 14.19, CG: 16.74, *p* = 0.001) (Table 4).

**Table 4:** Motivation performance in SIMS tests.

Category	Time	EG	CG	Two-Way MANOVA		
		M ± SD	M ± SD	Time effect	Group effect	Interaction effect
Intrinsic motivation	Pre	16.44 ± 5.91	14.76 ± 5.67	*F* = 16.82 *η^2^* = 0.21 *p* < 0.001	*F* = 4.97 *η^2^* = 0.07 *p* = 0.03	*F* = 0.11 *η^2^* = 0.002 *p* = 0.74
	Post	20.22 ± 3.20	17.97 ± 4.62			
Identified regulation	Pre	16.34 ± 4.96	14.76 ± 6.14	*F* = 14.96 *η^2^* = 0.19 *p* < 0.001	*F* = 4.48 *η^2^* = 0.07 *p* = 0.04	*F* = 0.17 *η^2^* = 0.003 *p* = 0.69
	Post	19.91 ± 3.86	17.65 ± 4.70			
External regulation	Pre	17.72 ± 6.42	20.18 ± 3.95	*F* = 13.23 *η^2^* = 0.17 *p* = 0.001	*F* = 9.20 *η^2^* = 0.13 *p* = 0.003	*F* = 0.002 *η^2^* < 0.001 *p* = 0.96
	Post	14.19 ± 5.15	16.74 ± 4.80			
Amotivation	Pre	16.56 ± 7.47	19.38 ± 4.29	*F* = 13.91 *η^2^* = 0.18 *p* < 0.001	*F* = 14.96 *η^2^* = 0.19 *p* < 0.001	*F* = 0.54 *η^2^* = 0.008 *p* = 0.47
	Post	12.09 ± 3.60	16.38 ± 5.93			

EG, experimental group; CG, control group; M, mean; SD, standard deviation.

## 4 Discussion

Participants improved basic sail knowledge after 6-week courses given by either VR sessions or traditional lectures. It is necessary to highlight the favorable outcomes associated with VR learning. EG showed a higher accuracy rate than CG in overall knowledge in the post-test, and the magnitude of improvement is significantly larger than CG. Specifically, VR technology facilitated understandings on sailing skills and navigation rules, which was substantiated by preferable performance of VR-based sessions over traditional lectures in the post-test. In addition to the differences between the teaching approaches, comparable performance was identified in terms of knowledge on sailboat structure. Therefore, the main findings provided critical insights into applying VR technology to course design and teaching.

The current study is consistent with a recent meta-analysis revealing that HMD-based immersive learning has an overall better effect on learning performance than non-immersive learning approaches (Wu et al., 2020). According to the experiential learning theory, “learning is the process whereby knowledge is created through the transformation of experience” (Kolb, 1984). The VR-based sail education program was designed in line with the four-step cycle of experiencing, reflecting, thinking, and acting. Authentic feeling was simulated by the first-person view, 3D modeling, spherical video, and virtual field trips (Weyhe et al., 2018; Chien et al., 2020; Han, 2020). A prominent advantage of VR-based lectures over traditional lectures is the concrete experience in learning. Additionally, VR-based lectures associated with demonstration in a virtual environment facilitated students making reflective connections between teaching content and observation. The reflective observation then helped the students to engage in thinking to comprehend concepts, form theories, and reach conclusions. After each VR-based lecture, self-regulated learning sessions were assigned which provided students with affordances of learning by doing. In this stage, the students tested their theories and thoughts through active experimentation, thus establishing direct connections between operations and outcomes. The enhanced learning effects can be also explained by embodied learning theory, which highlights the relationship between body movements and cognitive processes (Smith, 2024). Adding a motoric modality to the learning signal can activate more neural paths, which can make learning signals or memory tracking stronger (Kim et al., 2023).

An example that describes the learning process is the understanding of no-sail zone. “A sailboat can barely make any forward progress directly into the wind. When you tried to sail close to the wind, the sails simply flapped and you lost headway.” This interpretation is probably what students always heard about no-sail zone in traditional lectures. In the virtual environment, students are given the sailor role to trim the virtual sail and then can actually see how speed changes when the sailboat approaches, enters and leaves the zone. In terms of upwind sailing, the smaller the angle between the boat and the wind direction, the slower the speed. By observing the prominent change in speed, students can better understand the concept of no-sail zone. Therefore, explorations in the virtual environment enhance learning by doing.

Situational motivation refers to the motivation that an individual experiences while currently engaged in an activity (Vallerand, 1997). This concept provides valuable insights into an individual’s current self-regulation processes. To further refine and describe the situational motivation, self-determination theory (SDT) divides motivation into four components including intrinsic motivation (IM), identified regulation (IR), external regulation (ER) and amotivation (AM), which constitute a self-determination continuum from high to low levels (Deci and Ryan, 1991). The results obtained through the 7-point Likert scale in the current study showed that students’ IM and IR increased to a larger extent associated with VR sailing. On the other hand, greater reductions ER and AM were identified in EG compared to CG. Specifically, VR captured students’ inherent interest and enjoyment in learning about sailing, thereby enhancing their IM (Deci and Ryan, 2000). IM is a critical component of self-determination theory and is likely responsible for the preponderance of human learning across the lifespan (Bouffard et al., 2017). In formal education, IM has been shown to predict student engagement and, in turn, higher achievement (GPA) (Froiland and Worrell, 2016). Therefore, the increase in IM associated with VR sailing can be seen as a positive outcome that may lead to improved learning and engagement among students. The increased IR means that students consciously identified with or personally endorsed the value of the sailing activity and experienced a relatively high degree of volition or willingness to act (Ryan and Deci, 2020). IR is a form of motivation that is more autonomous and self-determined than ER. Therefore, the increase in IR associated with VR sailing can be seen as a positive outcome that may lead to more sustained engagement and effort among students. Notably, the study found that ER decreased in the EG compared to the CG. ER concerns behaviors driven by externally imposed rewards and punishments and is typically experienced as controlled and non-autonomous (Ryan and Deci, 2020). The significant decrease in ER in the EG suggests that VR was able to reduce the reliance on external rewards and punishments among students, which may lead to more autonomous and self-determined motivation. Additionally, the study also found that AM decreased in the EG compared to the CG. AM can result from a lack of felt competence to perform or a lack of value or interest in the activity. It has been shown to be a strong negative predictor of engagement, learning, and wellness (Ryan and Deci, 2020). The decrease in AM in the EG suggests that VR was able to address some of the underlying issues that may have contributed to AM among students, such as a lack of interest or competence in sailing. This, in turn, may lead to improved engagement and learning among students. In conclusion, these findings suggest that VR-based education can facilitate increasing students’ self-determination and lead to improved learning and engagement among students.

These results are significant for future PE programs, especially sailing education. By providing immersive and interactive learning experiences, VR can help students develop a strong foundation in sailing skills and knowledge. This can make the transition to real sailing smoother and more effective. In addition, the engaging and enjoyable nature of VR can stimulate students’ interest in sailing and motivate them to further explore and engage with the sport. Despite the promising results, the study has several limitations that should be noted. For example, the sample size of the study was relatively small, which may limit the generalizability of the findings to larger populations. Besides, the sail education program consisted of 12 sessions over 6 weeks. It is unclear whether the benefits of VR-based learning would persist over longer periods of time or with more extensive use of the technology. Furthermore, this study only explored the impact of VR on students’ theoretical sailing knowledge, which is an important starting point, but clearly not sufficient to fully assess the effectiveness of VR in the field of sailing education. Therefore, in future research, it is essential to combine VR-based learning with real-world practice to ensure that students develop comprehensive and practical sailing skills.

## 5 Conclusion

Sailing is considered effective in promoting health, life skills, self-esteem, and academic performance for children and adolescents. The current study compared VR lectures with traditional lectures in the effectiveness of students learning basic sail knowledge and motivation. Participants attending the VR lectures and self-regulated VR practice outperformed their counterparts taking traditional lectures in the post-test. Specifically, VR technology induced superior outcomes over traditional lectures in sailing skills, and navigation rules. An evidence-based conclusion can be reached that VR is an effective instrument to facilitate student novices learning basic sail knowledge. Compared with traditional lectures using sailboat model to introduce structure of a sailboat, VR sessions indicated no advantage. The finding implies that VR facilitates learning by helping the students to make observable and reflective connections between abstract knowledge and experience. Because VR could better improve students’ intrinsic motivation and identified regulation while better reduce external motivation and amotivation in sailing lessons. According to self-determination theory, it can be further deduced that VR-based sailing education facilitates increasing students’ self-determination. In conclusion, we suggest that VR is a promising supplement to traditional teaching methods, providing students with an active, engaging, and insightful learning experience. However, it cannot currently serve as a complete substitute for real-world sailing. This is because the existing VR technology is unable to replicate certain sensory elements, such as the feel of the wind, the smell of the sea, and the physical sensation of movement, all of which are important to the sailing experience.

## Data Availability

The original contributions presented in the study are included in the article/supplementary material, further inquiries can be directed to the corresponding author.

## References

[ref1] American Sailing Association (2010). Sailing made easy. Los Angeles, CA: American Sailing Association.

[ref2] Bouffard L. Ryan R. M. Deci E. L. (2017). Self-determination theory. Basic psychological needs in motivation, development and wellness. New York, NY: Guilford Press.

[ref3] Brookhart S. M. Nitko A. J. (2011). Educational assessment of students. 6th Edn. Boston: Allyn & Bacon.

[ref4] Capurso M. Borsci S. (2013). Effects of a tall ship sail training experience on adolescents’ self-concept. Int. J. Educ. Res. 58, 15–24. doi: 10.1016/j.ijer.2013.01.004

[ref5] Caraballo Vidal I. Pezelj L. Ramos-Álvarez J. J. Guillen-Gamez F. D. (2024). Level of satisfaction with the application of the collaborative model of the flipped classroom in the sport of sailing. Educ. Sci. 14:150. doi: 10.3390/educsci14020150

[ref6] Chien S.-Y. Hwang G.-J. Jong M. S.-Y. (2020). Effects of peer assessment within the context of spherical video-based virtual reality on EFL students’ English-speaking performance and learning perceptions. Comput. Educ. 146:103751. doi: 10.1016/j.compedu.2019.103751

[ref7] Cohen J. (1988). Behavioral sciences, economics, finance, Business & Industry, social sciences. 2nd Edn. New York: Routledge.

[ref8] Cotterill S. T. Brown H. (2018). An exploration of the perceived health, life skill and academic benefits of dinghy sailing for 9-13-year-old school children. J. Adventure Educ. Outdoor Learn. 18, 227–241. doi: 10.1080/14729679.2018.1424001

[ref9] Deci E. L. Ryan R. M. (1991). “A motivational approach to self: integration in personality” in Nebraska symposium on motivation. ed. Dienstbier R., Perspectives on motivation, vol. 38 (Lincoln: University of Nebraska press), 237–288. 2130258

[ref10] Deci E. L. Ryan R. M. (2000). The “what” and “why” of goal pursuits: human needs and the self-determination of behavior. Psychol. Inq. 11, 227–268. doi: 10.1207/S15327965PLI1104_01

[ref11] Faul F. Erdfelder E. Buchner A. Lang A.-G. (2009). Statistical power analyses using G*power 3.1: tests for correlation and regression analyses. Behav. Res. Methods 41, 1149–1160. doi: 10.3758/BRM.41.4.1149, PMID: 19897823

[ref12] Froiland J. M. Worrell F. C. (2016). Intrinsic motivation, learning goals, engagement, and achievement in a diverse high school. Psychol. Sch. 53, 321–336. doi: 10.1002/pits.21901

[ref13] Garris R. Ahlers R. Driskell J. E. (2002). Games, motivation, and learning: a research and practice model. Simul. Gaming 33, 441–467. doi: 10.1177/1046878102238607

[ref14] Gray R. (2017). Transfer of training from virtual to real baseball batting. Front. Psychol. 8:2183. doi: 10.3389/fpsyg.2017.02183, PMID: 29326627 PMC5733365

[ref15] Guay F. Vallerand R. J. Blanchard C. (2000). On the assessment of situational intrinsic and extrinsic motivation: the situational motivation scale (SIMS). Motiv. Emot. 24, 175–213. doi: 10.1023/A:1005614228250

[ref16] Han I. (2020). Immersive virtual field trips in education: a mixed-methods study on elementary students' presence and perceived learning. Br. J. Educ. Technol. 51, 420–435. doi: 10.1111/bjet.12842

[ref17] Hattie J. Marsh H. W. Neill J. T. Richards G. E. (1997). Adventure education and outward bound: out-of-class experiences that make a lasting difference. Rev. Educ. Res. 67, 43–87. doi: 10.3102/00346543067001043

[ref18] Ji F. Zhang X. Zhao S. Fang Q. (2023). Virtual reality: a promising instrument to promote sail education. Front. Psychol. 14:1185415. doi: 10.3389/fpsyg.2023.1185415, PMID: 37564315 PMC10410853

[ref19] Kim T. Planey J. Lindgren R. (2023). Theory-driven Design in Metaverse Virtual Reality Learning Environments: two illustrative cases. IEEE Trans. Learn. Technol. 16, 1141–1153. doi: 10.1109/TLT.2023.3307211

[ref20] Kolb D. (1984). Experiential learning: experience as the source of learning and development. Prentice-Hall.

[ref21] Lien A. J. (1971). Measurement and evaluation of learning. 2nd Edn. Dubuque, IA: William C. Brown.

[ref22] Mcculloch K. Mclaughlin P. Allison P. Edwards V. Tett L. (2010). Sail training as education: more than mere adventure. Oxf. Rev. Educ. 36, 661–676. doi: 10.1080/03054985.2010.495466

[ref23] Morales-Belando M. T. Arias-Estero J. L. (2017). Effect of teaching races for understanding in youth sailing on performance, knowledge, and adherence. Res. Q. Exerc. Sport 88, 513–523. doi: 10.1080/02701367.2017.1376032, PMID: 29048249

[ref24] Nunnally J. Bernstein I. (1994). Psychometric theory. 3rd Edn. New York: McGraw-Hill.

[ref25] Oagaz H. Schoun B. Choi M. H. (2022). Performance improvement and skill transfer in table tennis through training in virtual reality. IEEE Trans. Vis. Comput. Graph. 28, 4332–4343. doi: 10.1109/TVCG.2021.3086403, PMID: 34081582

[ref26] Østerlie O. Løhre A. Haugan G. (2019). The situational motivational scale (SIMS) in physical education: a validation study among Norwegian adolescents. Cogent Educ. 6:1603613. doi: 10.1080/2331186X.2019.1603613

[ref27] Plotzky C. Lindwedel U. Sorber M. Loessl B. König P. Kunze C.. (2021). Virtual reality simulations in nurse education: a systematic mapping review. Nurse Educ. Today 101:104868. doi: 10.1016/j.nedt.2021.104868, PMID: 33798987

[ref28] Qingdao G. Q. (2021). Five-year plan for sailing promotion (2021–2025). Qingdao Government.

[ref29] Roussou M. (2004). Learning by doing and learning through play: an exploration of interactivity in virtual environments for children. Comput. Entertain. 2:10. doi: 10.1145/973801.973818

[ref30] Ryan R. M. Deci E. L. (2020). Intrinsic and extrinsic motivation from a self-determination theory perspective: definitions, theory, practices, and future directions. Contemp. Educ. Psychol. 61:101860. doi: 10.1016/j.cedpsych.2020.101860

[ref31] Schmitt A. Atencio M. Sempé G. (2020). “You're sitting on a hot soccer field drinking Gatorade … I’m sitting in a yacht club just enjoying the view, enjoying the drinks”: parental reproduction of social class through school sport sailing. Eur. Phys. Educ. Rev. 26, 987–1005. doi: 10.1177/1356336X20911386

[ref32] Smith C. (2024). Embodied learning in a virtual mathematics classroom: an example lesson. Int. J. Math. Educ. Sci. Technol. 55, 1084–1095. doi: 10.1080/0020739X.2023.2197906

[ref33] Trinidad-Fernández M. Bossavit B. Salgado-Fernández J. Abbate-Chica S. Fernández-Leiva A. J. Cuesta-Vargas A. I. (2023). Head-mounted display for clinical evaluation of neck movement validation with Meta quest 2. Sensors 23. doi: 10.3390/s23063077 PMC10056752 36991788

[ref34] Tsai W. L. Pan T. Y. Hu M. C. (2022). Feasibility study on virtual reality based basketball tactic training. IEEE Trans. Vis. Comput. Graph. 28, 2970–2982. doi: 10.1109/TVCG.2020.3046326, PMID: 33351762

[ref35] Vallerand R. J. (1997). “Toward a hierarchical model of intrinsic and extrinsic motivation” in Advances in experimental social psychology. ed. Zanna M. P. (New York: Academic Press), 271–360.

[ref36] Weyhe D. Uslar V. Weyhe F. Kaluschke M. Zachmann G. (2018). Immersive anatomy atlas-empirical study investigating the usability of a virtual reality environment as a learning tool for anatomy. Front. Surg. 5:73. doi: 10.3389/fsurg.2018.00073, PMID: 30560134 PMC6284347

[ref37] Wu B. Yu X. Gu X. (2020). Effectiveness of immersive virtual reality using head-mounted displays on learning performance: a meta-analysis. Br. J. Educ. Technol. 51, 1991–2005. doi: 10.1111/bjet.13023

[ref38] Zhang F. Zhang B. Wang X. Huang C. Hu B. (2023). Effects of tai chi on insomnia in elderly people with chronic non-specific low back pain: a study protocol for a randomized controlled trial. Front. Psychol. 14:1105359. doi: 10.3389/fpsyg.2023.1105359 36910817 PMC9998706

